# Optimal physiotherapy management strategies for cervical dystonia: an international Delphi study

**DOI:** 10.1007/s10072-026-09314-8

**Published:** 2026-08-20

**Authors:** Melani J Boyce, Camila Quel de Oliveira, Alana B. McCambridge, Colleen G. Canning, Peter Stubbs, Arianne P. Verhagen

**Affiliations:** 1 Discipline of Physiotherapy, Graduate School of Health, University of Technology, Sydney, Australia; 2 Physiotherapy Department, Westmead Hospital, Sydney, Australia; 3 Discipline of Physiotherapy, School of Health Sciences, Faculty of Health, Western Sydney University, Sydney, Australia; 4 Person Centred Rehabilitation Research Centre, Auckland University of Technology, Auckland, New Zealand; 5 Faculty of Medicine and Health, The University of Sydney, Sydney, Australia

**Keywords:** Cervical dystonia, Physiotherapy, Assessment, Treatment

## Abstract

**Background:**

Isolated cervical dystonia is a rare, lifelong, focal, idiopathic dystonia causing uncontrollable spasms in the neck. In conjunction with medical management, physiotherapy can improve pain and quality of life, however there are no evidence-based guidelines indicating optimal physiotherapy goal setting, assessments and treatments.

**Purpose:**

To determine international consensus among physiotherapists who regularly treat people with cervical dystonia on optimal physiotherapy management.

**Methods:**

A Delphi study was conducted between June 2024 and September 2025. Physiotherapists with more than 12 months experience working with people with cervical dystonia were invited to participate. The first survey asked participants to rate how essential common physiotherapy assessment and treatment techniques were for the management of people with cervical dystonia on a 5-point Likert scale (strongly agree to strongly disagree). Responses were analysed descriptively with a 70% threshold indicating agreement between participants. The second survey clarified if the agreed physiotherapy techniques were “essential” or “optional”.

**Results:**

Ten physiotherapists completed the first survey, and nine completed the second. Participants were mostly female (*n* = 6) and from Europe (*n* = 7). Essential assessments included head and neck position at rest, posture, tremor, movement, pain and impact on quality of life. Essential treatments included education, postural correction, active neck exercises, relaxation techniques, sensory retraining and other treatments relating to specific individual goals.

**Conclusion:**

This Delphi study achieved consensus on recommendations for physiotherapy assessment, goal setting and treatment of cervical dystonia which reflect expert opinion and can be used in the future in controlled trials to enable the development of clinical guidelines.

**Supplementary Information:**

The online version contains supplementary material available at https://doi.org/10.1007/s10072-026-09314-8.

## Introduction

Isolated cervical dystonia (CD) is a focal, idiopathic dystonia causing uncontrollable spasms in the muscles of the neck and upper back [1, 2]. CD is a rare, lifelong condition with a prevalence of 9.95 per 100,000, (95% Confidence Interval (CI): 3.51–28.17) [3] which generally presents around 40 years of age [4, 5]. Non-motor impairments such as neck pain, depression and anxiety are commonly seen in CD, affecting all aspects of a person’s life [6–10].

Botulinum toxin injections into the dystonic muscles temporarily reduce the motor and non-motor impairments of CD, improving pain and quality of life [11–13]. When provided in conjunction with injections, physiotherapy can further improve pain and quality of life. A recent systematic review of 14 studies (8 randomised controlled trials (RCTs), 3 case-controlled studies and 3 case-series) assessed the effectiveness of physiotherapy in the management of CD [14]. Treatments were mostly applied in multimodal protocols including (but not limited to) stretching, exercise therapy, strengthening, motor learning, neck mobilisations, taping and biofeedback. Treatment duration and frequency varied across studies and 11/14 studies combined physiotherapy with botulinum toxin injections. The review included a meta-analysis of two studies [15, 16] that showed physiotherapy in addition to botulinum toxin reduced pain measured on the Toronto Western Spasmodic Torticollis Rating Scale (TWSTRS) pain scale. Seven studies reported that physiotherapy improved disease severity, disability and quality of life, however the heterogeneity of treatment type and duration meant recommendations were uncertain [14]. A second systematic review of multimodal treatments for CD included 13 studies (7 RCTs, 3 cross-over designs, 2 case-series and 1 pre-post trial). No meta-analysis was performed due to the heterogeneity of treatment protocols across the studies. The authors concluded that physiotherapy is an effective addition to botulinum toxin [17]. Other reviews have found identifying effective physiotherapy interventions or combinations of interventions difficult due to the small number of clinical trials, small sample sizes, low methodological quality, and varied treatment parameters [8, 18, 19]. All reviews have highlighted the need for further high-quality controlled trials to enable the establishment of evidence-based recommendations on the optimal physiotherapy management of CD.

Despite the lack of evidence to inform guidelines for physiotherapy management of CD, people with CD report that physiotherapy is beneficial in reducing their symptoms and many seek out physiotherapy treatment. A recent survey of 128 people with CD reported that 66% had tried physiotherapy to help manage their symptoms [20] and 66% of these found physiotherapy improved their ability to move their neck with ease and 55% reported reduced pain due to CD. Another recent survey of 91 people with CD found that of the 54% of participants who had physiotherapy the majority reported reduced pain [21]. Considering CD is a rare condition, many physiotherapists may have limited experience treating people with CD. Guidance for physiotherapists regarding physiotherapy management and useful treatment modalities for CD is required.

Given the limited and uncertain evidence of the effectiveness of physiotherapy interventions from controlled trials, the Delphi method was selected to systematically gather expert consensus to facilitate and guide clinical decision-making in the absence of formal guidelines. Identifying the assessment and treatment strategies most frequently used by physiotherapists who regularly manage CD is a critical first step toward developing clinical guidelines to inform future clinical management and research. Therefore, this study aims to determine international consensus among physiotherapists who regularly treat people with CD on which physiotherapy goals, assessment and treatment strategies can be recommended in the management of CD.

## Methods

### Study design

This Delphi study was conducted between June 2024 and September 2025. The Delphi approach is an anonymous consensus-based research method that consists of a series of structured questionnaires over multiple rounds to gain consensus among expert participants on a certain topic. Responses from earlier rounds are summarised in later rounds. Rounds continue to a predefined maximum, or until consensus is reached [22]. This study was approved by the University of Technology Sydney (UTS) Human Research Ethics Committee (ETH23-8851). Results are presented in accordance with the DELPHISTAR (COREQ) recommendations for standardized reporting of Delphi studies in Health sciences [23].

### Participants

Eligible participants were international expert physiotherapists who work in the management of people with CD. Sixty invitations were sent via email. Potential participants were identified through published research on dystonia, national and international dystonia associations (e.g., Dystonia Network Australia and Dystonia Europe), physiotherapy associations (e.g., the Australian Physiotherapy Association and the American Physical Therapy Association), through internet searches, and from the authors’ professional networks. Potential participants were encouraged to forward the invitation to others who may be eligible to join the study. Participants were eligible if they were a qualified physiotherapist, were able to complete the survey in English, and had more than 12 months experience researching or providing physiotherapy care to people with CD. People who were part of the authorship team were excluded from participation.

### Procedure

Invitations and advertisements for the study contained a QR code and link to the first survey where potential participants could provide informed consent to participate. All consenting participants answered eligibility questions, and if deemed eligible, progressed to the first round of survey questions. The survey was conducted online using Qualtrics software. Participants were contacted via email throughout the study, with reminders provided one week before the closing date if required, and each survey remained open for four weeks.

### Development of survey 1

The development of the first survey was guided by findings from a literature search of physiotherapy assessment and treatment techniques that had been used in studies involving people with CD [15, 16, 24–40]. Additional items were identified using the authors’ clinical experience, particularly related to goals of physiotherapy, frequency and duration of treatment and measurement tools.

### Round 1 survey

The first survey had two parts; (1) demographic information including years working as a physiotherapist or researcher, years working with people with CD, percentage of workload spent managing people with CD, country of practice and gender, and (2) ratings on how essential each assessment, measurement tool, treatment technique and goal of physiotherapy was for managing people with CD using a five point Likert scale from “strongly agree” to “strongly disagree”. Participants were provided the opportunity to add information on their experiences of managing people with CD through open-ended questions embedded throughout the survey. The open-ended responses were analysed and incorporated in the subsequent Delphi round.

The results of survey 1 were analysed descriptively, and agreement was assessed between participants, with an a priori defined 70% threshold indicating agreement (agree and strongly agree responses).

### Round 2 survey

In the second survey, participants were provided the full results of the previous survey, a summary identifying which items had reached consensus as essential and a link to the second survey questions. As there were many goals, assessment and treatment techniques that met the threshold for agreement by participants in the round 1 survey, we decided to ask participants to reconsider these in the round 2 survey and indicate whether they were “essential” or “optional” components of care. Participants were also asked to rank the goals of treatment that had reached the threshold for agreement from most to least important in their management of people with CD.

After round 2, a summary of the physiotherapy management tools (relating to assessment, measurement, goals and treatment) for CD that had reached 70% agreement was shared with participants for approval. Survey questions for all rounds are provided in the Online Resources 1 and 3.

## Results

### Participants

**Fig. 1:**
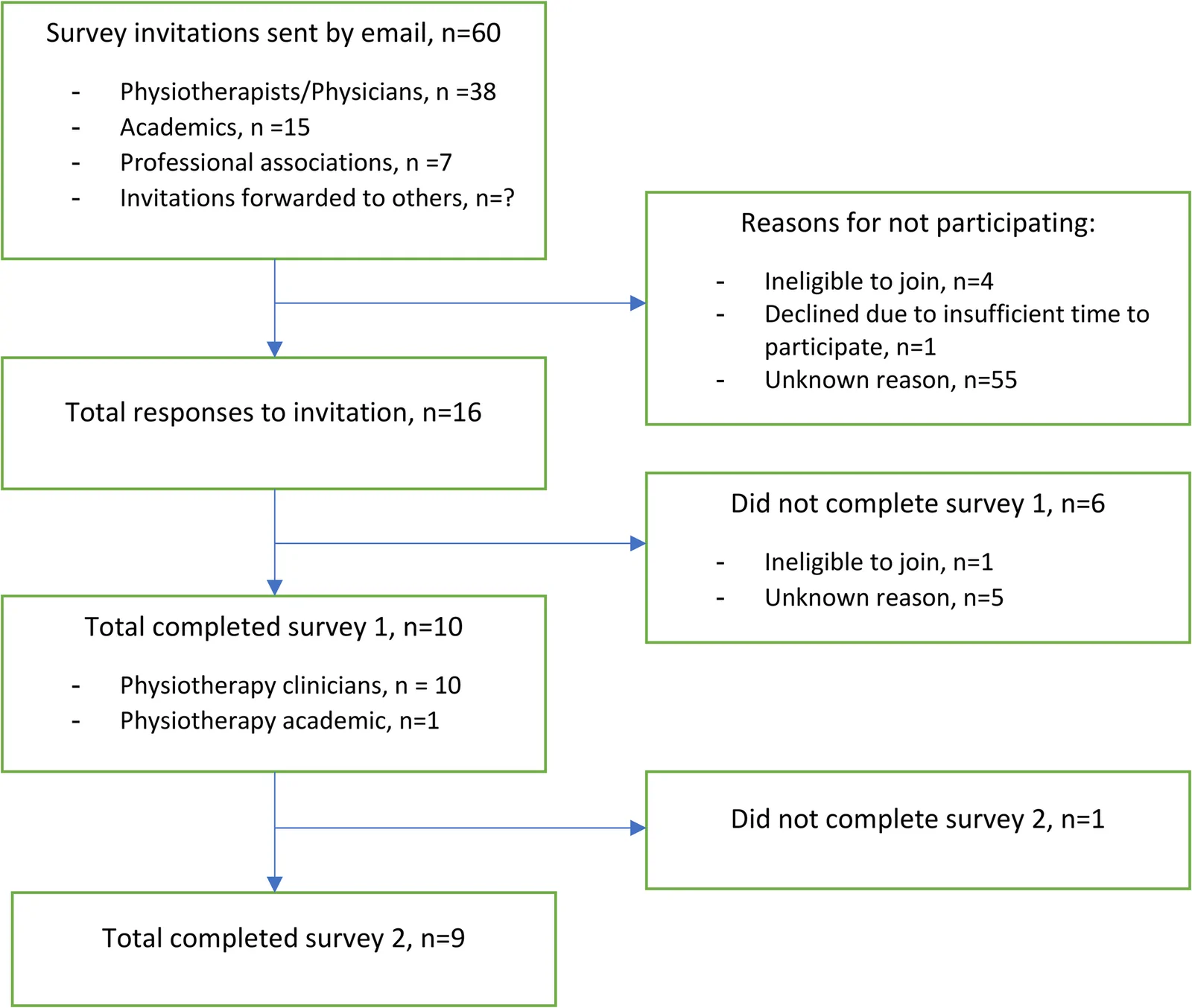
Flow of participants through the study

From 60 invitations, 10 physiotherapists completed the first survey round, and nine the second survey round (Fig. 1). Agreement was obtained in the second round on all survey items therefore no further surveys were needed. The demographic data of participants is shown in Table 1.

**Table 1:** Demographic information of participants

	Survey 1 (*n* = 10)	Survey 2 (*n* = 9)
Gender	Female *n* = 7 (70%)	Female *n* = 6 (67%)
Time worked as a physiotherapist (years)	> 11 years − 10 (100%)	> 11 years − 9 (100%)
Country of work	The Netherlands – 3 (30%) Australia – 2 (20%) UK – 2 (20%) France − 1 (10%) Belgium – 1 (10%) USA – 1 (10%)	The Netherlands – 3 (33%) Australia – 1 (11%) UK – 2 (22%) France − 1 (11%) Belgium – 1 (11%) USA – 1 (11%)
Type of work – clinical/academic	Clinical physiotherapist – 10 (100%)	Clinical Physiotherapist – 9 (100%)
Time worked with people with CD	< 5 years – 2 (20%) 6–10 years – 6 (60%) > 11 years – 2 (20%)	< 5 years – 2 (22%) 6–10 years – 5 (56%) > 11 years – 2 (22%)
Amount of work time spent working with CD (%)*		1–25% − 4 (44%) 26–50% − 2 (22%) 51–75% − 1 (11%) 76–100% − 2 (22%) Range 1% − 100% Average = 40%

UK – United Kingdom; USA – United States of America; CD – cervical dystonia

*Question asked in survey 2 only

### Findings

The recommendations for physiotherapy management of people with CD are shown in Table 2. The complete results are included in the Online Resources 2 and 4.

**Table 2:** Recommendations for physiotherapy management of CD

Essential items to perform	Optional items to be performed as required
**Assessment** • Assess pain due to CD • Assess difficulty of working or performing self-care and household tasks (e.g. personal hygiene, house and garden chores, driving, working etc.) • Ask about neurotoxin use (e.g. type, dosage, frequency of injection and effect) • Ask about other medical treatments used to manage CD (e.g. oral medication, deep brain stimulation) • Ask about other medical conditions which may impact on CD • Observe the dystonic position of the head (head turn) and muscle over-activity • Identify which muscles are dystonic (over-active) • Observe presence/absence of head or limb tremor • Observe posture of the neck and spine • Assess the ability to actively correct head position and maintain the aligned position for a short time • Assess neck and upper limb AROM • Assess neck and upper limb PROM^ • Assess ability to coordinate movements of the head, neck and upper limbs	• Palpation of the neck, shoulders and upper back • Assess neck, upper and lower limb muscle strength • Assess balance • Assess walking speed and ability • Assess any recent falls and ongoing falls risk • Assess exercise tolerance and participation in regular exercise • Assess vision
**Measurement of impairments** • VAS for pain	• Manual muscle testing for muscle strength of the neck upper and lower limbs • A standardized scale for measuring CD severity, e.g. the TWSTRS • A standardized scale for measuring the impact of CD on quality of life, e.g. the CDQ-24 or CDIP-58 • Any standardized measure of functional tasks including walking or balance, e.g. MiniBEST, FGA, Falls assessment scales
**Goals of physiotherapy (in order of importance)** Treatment goals should be created with the person with CD addressing specific needs that encourage self-management. • To teach self-management strategies for CD • To improve active head control at rest and during movement • To reduce pain related to CD • To improve / increase active range of movement of the neck • To improve sensorimotor integration • To improve general fitness and increase participation in general exercise • To improve strength in the muscles of the neck and upper back • To improve / reduce head, neck or upper limb tremor • To improve strength, coordination and function of the upper limbs • To improve walking function (e.g. gait pattern, speed, safety) • To improve balance	
**Treatment techniques** Frequency, duration and timing of treatment should be determined on a case-by-case basis depending on duration and severity of symptoms, the person’s goals and their response to treatment. • Education on CD • Active movement exercises for the neck and/or upper limbs • Exercises and education to improve posture • Relaxation techniques (e.g. Progressive muscle relaxation) • Increasing participation in general physical activity (e.g. swimming, walking, playing sport etc.) • Sensory retraining • Other treatment techniques that address specific individual goals of the person with CD **Treatment techniques NOT recommended:** • Cold/ice packs – may increase CD symptoms temporarily	• Education on meditation and/or mindfulness techniques • Exercises to improve upper limb movement and function • Breathing exercises • Passive muscle stretches for the neck, upper limbs and back • Strengthening exercises for neck, upper back and upper limb • Gait / walking retraining • Balance exercises / re-training • Visual tracking tasks / exercises to encourage improved range and quality of neck movement **Treatment techniques to use with caution:** • Massage to the neck, upper back and shoulder - may increase CD symptoms temporarily but may also improve pain - effects should be monitored closely

CD – cervical dystonia; AROM – active range of motion; PROM – passive range of motion; VAS – Visual analogue scale; TWSTRS – Toronto Western Spasmodic Torticollis Rating Scale; CDQ-24 – Cervical Dystonia Questionnaire 24; CDIP-58 – Cervical Dystonia Impact Profile 58; MiniBEST – Mini Balance Evaluation Systems Test; FGA – Functional Gait Assessment; ^ authors designated as essential based on one participant’s feedback

#### Physiotherapy assessment

Participants agreed that the physiotherapy assessment of people with CD should include a detailed history documenting pain, problems doing usual tasks (for example, personal hygiene, work, household chores and leisure activities), concurrent medical conditions, current medical management, and botulinum toxin use. Assessments should include observation of head and neck position, posture, muscles affected by dystonia, tremor and active and passive movements of the head and upper body. Optional assessments include strength of the neck and upper limbs, walking ability, balance, falls, exercise tolerance and vision (Table 2).

Measurement of impairments were considered essential by participants, however the range of outcome measures used by practitioners was broad, making it difficult to achieve consensus on any specific outcome measures except for the visual analogue scale (VAS) for pain which was considered essential. Participants agreed that standardised measures for CD (for example the Toronto Western Spasmodic Torticollis Scale) and standardised measures for functional tasks, like balance and walking, were optional (Table 2).

#### Management goals

Participants agreed that the goals of physiotherapy should be individualised and determined in consultation with the person with CD to encourage self-management. Goals of physiotherapy that were deemed essential were ranked by participants in order of importance. Physiotherapy goals that were considered the most important include improving head control, reducing pain, improving neck active movement and sensorimotor integration. Less important goals included improving exercise participation and fitness, strength in neck and back muscles, walking and balance (Table 2).

#### Physiotherapy management

Treatment techniques that were deemed essential include education on CD and postural correction, active exercises for the neck and upper limbs, relaxation techniques, sensory retraining and other treatments relating to specific individual goals. Encouraging increased participation in general exercise was also deemed essential (Table 2).

Optional treatment techniques include stretches and strengthening exercises for the neck and upper body, gait, balance and coordination retraining. Participants did not recommend that ice or cold packs to be used for neck pain as they may lead to a temporary worsening of symptoms. Participants also agreed that massage to the neck, shoulders, and upper back should be used with caution as it may lead to a temporary worsening of symptoms for some, while others may find it helpful for pain management. Finally, participants agreed that the timing, frequency, and duration of physiotherapy treatment should be determined individually with the person with CD depending on their severity of symptoms, their goals and response to treatment (Table 2).

## Discussion

This Delphi study established international expert consensus on current physiotherapy assessment, goals and treatment strategies for managing CD. Participants deemed it essential to assess head and neck position at rest, posture, tremor, movement, pain and the impact of CD on quality of life. Muscle strength and functional ability including walking, balance, exercise tolerance and falls were considered optional assessments, when relevant. Measurement of pain using a VAS was considered essential, while standardised measures for CD severity, functional activities and quality of life were considered optional. The person with CD should be involved in setting goals which encourage self-management and may aim to improve head control, pain, neck movement and strength, sensorimotor integration, general exercise, walking and balance. Treatment techniques deemed essential include education on CD, postural correction, active neck exercises, relaxation techniques, sensory retraining and encouraging increased exercise participation. Functional retraining of walking, balance and fall prevention may be employed as indicated by assessment findings. Cold packs are not recommended, and massage should be used with caution, as a temporary worsening in symptoms may occur.

Previous controlled trials investigating physiotherapy interventions for people with CD have employed some of the assessment and treatment techniques recommended in this study. For example, multiple studies used active exercises and stretches for treatment of people with CD [24, 26, 31]. Most studies used multi-modal treatment [15, 16, 26] making it difficult to know the effectiveness of individual techniques. A recent systematic review suggests that physiotherapy for cervical dystonia should include neck active movements, muscle strengthening, stretching and relaxation of over-active muscles [17], which are all techniques that have been recommended in this study by our participants. Guidance videos that suggest specific exercises for people with CD [37] and a website *(*Dystonia Physio Exercise Hub*)* [41] have been created but have not yet been assessed in controlled trials. The current study provides much needed comprehensive additional information to these resources and can be used as a guide for management of people with dystonia clinically and in research studies.

The expert physiotherapists surveyed in this study agreed that people with CD should be fully involved in their individualised physiotherapy management with a self-management focus. Many of the recommended techniques involve education and regular exercise, which can be incorporated into daily routines of people with CD with the goal of improving health and quality of life. Consistent with other studies, physiotherapists managing people with CD are encouraged to support overall wellness by promoting good health behaviours, such as increased physical activity, in order to improve quality of life [42]. By also addressing the non-motor features of CD (for example pain, fatigue, poor sleep, anxiety and depression), a holistic management approach could have a broader effect on quality of life for people with CD [10, 43].

Surveys of people living with CD also support a multimodal approach to management. In addition to medical treatments like botulinum toxin, people with CD report the most useful ways to manage their symptoms include physiotherapy and participation in general exercise, alongside self-management strategies such as hot packs, relaxation, and maintaining work, social and leisure activities [20]. Recent interviews with people with CD show that many are employing coping strategies such as prioritizing important life tasks, maintaining an active lifestyle and accepting a new life situation with a chronic condition to better manage their dystonia [43]. Our study supports these findings by recommending that all treatment goals be individualised and determined in consultation with the person with CD, encouraging a person-centred self-management approach.

### Study limitations

A potential limitation of this study is the small sample size. Due to the rarity of the condition, physiotherapists are unlikely to be treating people with CD unless they are associated with a neurology clinic offering concurrent specialised medical management, usually located in cities or large regional centres. This limits the number of physiotherapists exposed to people with CD, reducing the number of available “experts” for a Delphi study. Another limitation was that there were no participants from Asia or Africa, and the survey was only conducted in English. Efforts were made to engage physiotherapists in these regions, with advertisements in English sent to physiotherapy and dystonia associations in Africa and Asia, however, non-English speaking physiotherapists could not participate. Most participants were from Europe, which reflects where most of the literature, published in English, originates. With only 10 participants in round 1, and most based in Europe, this provides a limited view on how CD is managed globally.

It is possible that asking participants in the round 2 survey to reconsider the large number of items that reached consensus in the round 1 survey could have introduced bias into the results. It is possible that some items which did not reach consensus in round 1, may have reached consensus in round 2 if they were included in the second survey. However, this was a pragmatic decision made to streamline the final recommendations to ensure they are useful for physiotherapists in their clinical practice.

### Implications for clinical practice and future research

In the absence of clinical guidelines for the physiotherapy management of people with CD, the opinions of experts gathered in this Delphi study make an important contribution by guiding current clinical decision making and directing future research for CD. The recommendations made regarding goals, assessment and treatment techniques for people with CD can be used to inform the development of a clinical practice guideline on the physiotherapy management of CD, which in turn, can improve the care provided to this population.

Future research could investigate the effect of a holistic person-centred management approach which includes botulinum toxin injections, physiotherapy, management of non-motor symptoms and self-management strategies to investigate if better outcomes for people with CD can be achieved. Co-designing trials with people with CD will be an important step towards the development of management approaches that are practical and useful for those who will use them [44, 45].

## Conclusion

In the absence of evidence-based guidelines, we reached consensus on expert-based recommendations for the physiotherapy assessment, goal setting and treatment of people with CD through a Delphi process. While these recommendations offer valuable guidance, they should be interpreted as expert opinion rather than definitive clinical guidelines until validated through future controlled trials.

## Supplementary Information

Below is the link to the electronic supplementary material.

Supplementary Material 1

## Data Availability

All data generated or analysed during this study are included in this published article and its supplementary information files provided as online resources.
