# Supplementary material for: Optimal physiotherapy management strategies for cervical dystonia: an international Delphi study
Source: Neurol Sci. 2026 Aug 20;47(9):720. doi: 10.1007/s10072-026-09314-8 (PMC13490222; doi:10.1007/s10072-026-09314-8)
Supplement: Supplementary file 1 [file 10072_2026_9314_MOESM1_ESM.pdf]

Optimal physiotherapy management strategies for cervical dystonia: An International Delphi study

Neurological Sciences

Online resources

Boyce MJ, Quel de Oliveira C, McCambridge AB, Bradnam LV, Canning CG, Stubbs P, Verhagen AP

Online Resource 1: Round 1 survey Questions

Eligibility questions:

1. Are you a qualified physiotherapist? Y/N (If N, go to end of survey)
2. Are you a physiotherapist with at least 12 months experience in treating people with Cervical dystonia? Y/N
3. Are you a physiotherapy academic with at least 12 months experience in researching cervical dystonia? Y/N
   If No to both, go to end

Demographic questions:

4. How many years have you worked as a physiotherapist in any area?
   a. Less than 5 years
   b. 6 -10 years
   c. More than 11 years

5. How many years have you been treating people with cervical dystonia or researching cervical dystonia?
   a. Less than 5 years
   b. 6 – 10 years
   c. More than 11 years

6. Please indicate your gender
   a. Female
   b. Male
   c. Non-binary
   d. Prefer not to specify

7. Which country do you work in?

Assessment of people with cervical dystonia – Impairments

All statements are regarding a new patient with cervical dystonia. How much do you agree with the following statements?

1. It is essential to observe/assess the dystonic position of the head (head turn) and muscle over-activity
    a. Strongly agree
    b. Agree
    c. Neither agree nor disagree
    d. Disagree
    e. Strongly disagree

2. What instruments do you use to assess/measure the dystonic position of the head/head turn? (for example, observation, palpation, photographs, video, Toronto Western Spasmodic Rating Scale (TWSTRS), TSUI scale, other scale etc). Please specify or answer "not applicable" (N/A).

3. It is essential to observe/assess/identify which muscles are dystonic (over-active)?
    a. Strongly agree
    b. Agree
    c. Neither agree nor disagree
    d. Disagree
    e. Strongly disagree

4. What instrument do you use to assess/measure the dystonic muscles? (for example, observation, palpation, Active or passive range of motion, muscle tone measurement, neck muscle strength, photographs, video, EMG etc). Please specify or answer "not applicable" (N/A).

5. It is essential to observe the presence/absence of head or limb tremor
    a. Strongly agree
    b. Agree
    c. Neither agree nor disagree
    d. Disagree
    e. Strongly disagree

6. What instrument do you use to assess/measure the head/neck tremor? (for example, observation, palpation, photographs, video, EMG etc). Please specify or answer "not applicable" (N/A).

7. It is essential to assess the patient's ability to actively correct their head position and maintain the aligned position for a period of time
    a. Strongly agree
    b. Agree
    c. Neither agree nor disagree
    d. Disagree
    e. Strongly disagree

8. It is essential to assess the patient's pain due to cervical dystonia
    a. Strongly agree
    b. Agree
    c. Neither agree nor disagree
    d. Disagree
    e. Strongly disagree

9. What instruments do you use to assess their pain? (for example, Visual Analogue Scale (VAS), Numerical Rating Scale (NRS) etc.). Please specify or answer "not applicable" (N/A).

10. It is essential to assess their neck and upper limb active range of movement
    a. Strongly agree
    b. Agree
    c. Neither agree nor disagree
    d. Disagree
    e. Strongly disagree

11. What instruments do you use to measure the neck active range of movement? (for example, inclinometer, goniometer, observation etc.). Please specify or answer "not applicable" (N/A).

12. What instruments do you use to measure the upper limb active range of movement? (for example, inclinometer, goniometer, observation etc.). Please specify or answer "not applicable" (N/A).

13. It is essential to measure their neck and upper limb passive range of motion
    a. Strongly agree
    b. Agree
    c. Neither agree nor disagree
    d. Disagree
    e. Strongly disagree

14. What instruments do you use to measure the neck passive range of movement? (for example, inclinometer, goniometer, observation etc). Please specify or answer "not applicable" (N/A).

15. What instruments do you use to measure the upper limb passive range of movement? (for example, inclinometer, goniometer, observation etc). Please specify or answer "not applicable" (N/A).

16. It is essential to measure neck and upper limb muscle strength
    a. Strongly agree

b. Agree
c. Neither agree nor disagree
d. Disagree
e. Strongly disagree

17. What instruments do you use to measure the neck and upper limb muscle strength (for example, manual muscle strength grading assessment using grades 1-5; other device etc). Please specify or answer “not applicable” (N/A).

18. It is essential to measure lower limb muscle strength
    a. Strongly agree
    b. Agree
    c. Neither agree nor disagree
    d. Disagree
    e. Strongly disagree

19. What instruments do you use to measure lower limb muscle strength (for example, manual muscle strength grading assessment using grades 1-5; other device etc). Please specify or answer “not applicable” (N/A).

20. It is essential to observe the posture of the neck and spine
    a. Strongly agree
    b. Agree
    c. Neither agree nor disagree
    d. Disagree
    e. Strongly disagree

21. What instruments do you use to measure posture of the neck and spine? Please specify or answer “not applicable” (N/A).

22. It is essential to assess their ability to coordinate movements of their head, neck and upper limbs
    a. Strongly agree
    b. Agree
    c. Neither agree nor disagree
    d. Disagree
    e. Strongly disagree

23. What instruments do you use to measure coordination of the head, neck and upper limbs? (for example, finger to nose testing, rapid alternating movements of upper limbs, movements with eyes closed etc). Please specify or answer “not applicable” (N/A).

24. It is essential to assess their ability to coordinate movements of their lower body?

a. Strongly agree
b. Agree
c. Neither agree nor disagree
d. Disagree
e. Strongly disagree

25. What instruments do you use to measure coordination of the lower body? (for example, lower limb coordination tests, rapid alternating movements, movements with eyes closed etc). Please specify or answer “not applicable” (N/A).

26. It is essential to assess their upper limb proprioception?

a. Strongly agree
b. Agree
c. Neither agree nor disagree
d. Disagree
e. Strongly disagree

26. What instruments/tests do you use to measure upper limb proprioception? Please specify or answer “not applicable” (N/A).

27. It is essential to assess their lower limb proprioception?
    a. Strongly agree
    b. Agree
    c. Neither agree nor disagree
    d. Disagree
    e. Strongly disagree
28. What instruments/tests do you use to measure lower limb proprioception? Please specify or answer “not applicable” (N/A).

29. It is essential to assess their vision?
    a. Strongly agree
    b. Agree
    c. Neither agree nor disagree
    d. Disagree
    e. Strongly disagree

30. What instruments/tests do you use to measure their vision? Please specify or answer “not applicable” (N/A).

Assessment - Activity Limitations and Participation Restrictions

31. It is essential to assess their balance?

a. Strongly agree
b. Agree
c. Neither agree nor disagree
d. Disagree
e. Strongly disagree

32. What instruments do you use to measure balance? (for example, single leg stance time, tandem stance/walk time, a standardised balance assessment scale) Please specify or answer “not applicable” (N/A).

33. It is essential to assess their walking speed and ability?
    a. Strongly agree
    b. Agree
    c. Neither agree nor disagree
    d. Disagree
    e. Strongly disagree

34. What instruments do you use to measure walking ability and speed? (for example, 10 meter walk speed, 6 minute walking test, Timed Up and Go (TUG), video etc.). Please specify or answer “not applicable” (N/A).

35. It is essential to assess any recent falls and ongoing falls risk?
    a. Strongly agree
    b. Agree
    c. Neither agree nor disagree
    d. Disagree
    e. Strongly disagree

36. What instruments do you use to measure falls or falls risk? (for example, a standardised falls assessment scale). Please specify or answer “not applicable” (N/A).

37. It is essential to ask about their exercise tolerance and participation in regular exercise?
    a. Strongly agree
    b. Agree
    c. Neither agree nor disagree
    d. Disagree
    e. Strongly disagree

38. What instruments do you use to assess exercise tolerance and participation in regular exercise? Please specify or answer “not applicable” (N/A).

39. It is essential to assess their difficulty working or performing self-care and household tasks (for example, personal hygiene, house and garden chores, driving, working etc)

a. Strongly agree
b. Agree
c. Neither agree nor disagree
d. Disagree
e. Strongly disagree

40. What instruments do you use to measure their difficulty working, performing self-care or household tasks? (for example, Cervical dystonia Questionnaire 24 (CDQ-24), Cervical Dystonia Impact Profile (CDIP-58) etc). Please specify or answer “not applicable” (N/A).

41. It is essential to ask about their neurotoxin use (for example, the neurotoxin type, dosage, frequency of injection and effect)?
    Strongly agree
    Agree
    Neither agree nor disagree
    Disagree
    Strongly disagree

42. It is essential to ask about other medical treatments used to manage their dystonia (for example oral medication, deep brain stimulation)?
    a. Strongly agree
    b. Agree
    c. Neither agree nor disagree
    d. Disagree
    e. Strongly disagree

43. It is essential to ask about other medical conditions which may impact on their cervical dystonia?
    a. Strongly agree
    b. Agree
    c. Neither agree nor disagree
    d. Disagree
    e. Strongly disagree

44. Are there any other impairments, activity limitations or participation restrictions that you feel are essential to assess in a new patient with cervical dystonia? Please specify.

Measurement of cervical dystonia severity

All statements are regarding any patient with cervical dystonia. How much do you agree with the following statements?

1. It is essential to measure cervical dystonia severity using a standardised scale, for example the Toronto Western Spasmodic Torticollis Rating Scale (TWSTRS) or TSUI scale

    a. Strongly agree
    b. Agree
    c. Neither agree nor disagree
    d. Disagree
    e. Strongly disagree

2. Please specify the scale you use most often or answer “not applicable” (N/A)

3. It is essential to use a standardised scale to measure the impact of cervical dystonia on a person’s quality of life (CD-24, CDIP-58 etc)?
    a. Strongly agree
    b. Agree
    c. Neither agree nor disagree
    d. Disagree
    e. Strongly disagree

4. Please specify the standardised scale you use most often or answer “not applicable” (N/A)

Goals of Physiotherapy treatment of cervical dystonia

All statements are regarding any patient with cervical dystonia. How much do you agree with the following statements?

The goal of physiotherapy treatment is to:

1. Permanently correct the patient’s resting head position to the normal alignment
    a. Strongly agree
    b. Agree
    c. Neither agree nor disagree
    d. Disagree
    e. Strongly disagree

2. Temporarily correct the patient’s resting head position to the normal alignment
    a. Strongly agree
    b. Agree
    c. Neither agree nor disagree
    d. Disagree
    e. Strongly disagree

3. Improve the patient’s active head control at rest and during movement
    a. Strongly agree
    b. Agree
    c. Neither agree nor disagree
    d. Disagree

e. Strongly disagree

4. Improve or reduce head, neck or upper limb tremor
    a. Strongly agree
    b. Agree
    c. Neither agree nor disagree
    d. Disagree
    e. Strongly disagree

5. Reduce the pain related to cervical dystonia
    a. Strongly agree
    b. Agree
    c. Neither agree nor disagree
    d. Disagree
    e. Strongly disagree

6. Improve the patient's sensorimotor integration
    a. Strongly agree
    b. Agree
    c. Neither agree nor disagree
    d. Disagree
    e. Strongly disagree

7. Improve or increase active range of movement of the neck
    a. Strongly agree
    b. Agree
    c. Neither agree nor disagree
    d. Disagree
    e. Strongly disagree

8. Improve passive range of movement of the neck
    a. Strongly agree
    b. Agree
    c. Neither agree nor disagree
    d. Disagree
    e. Strongly disagree

9. Improve strength in the muscles of the neck and upper back
    a. Strongly agree
    b. Agree
    c. Neither agree nor disagree
    d. Disagree
    e. Strongly disagree

10. Improve strength, coordination and function of the upper limbs
    a. Strongly agree
    b. Agree
    c. Neither agree nor disagree
    d. Disagree
    e. Strongly disagree

11. Improve or reduce stiffness of the facet joints of the neck
    a. Strongly agree
    b. Agree
    c. Neither agree nor disagree
    d. Disagree
    e. Strongly disagree

12. Improve general fitness and increase participation in general exercise
    a. Strongly agree
    b. Agree
    c. Neither agree nor disagree
    d. Disagree
    e. Strongly disagree

13. Improve the patient's balance
    a. Strongly agree
    b. Agree
    c. Neither agree nor disagree
    d. Disagree
    e. Strongly disagree

14. Improve walking function (for example gait pattern, speed, safety)
    a. Strongly agree
    b. Agree
    c. Neither agree nor disagree
    d. Disagree
    e. Strongly disagree

15. Teach self-management strategies for cervical dystonia
    a. Strongly agree
    b. Agree
    c. Neither agree nor disagree
    d. Disagree
    e. Strongly disagree

16. Reduce EMG activity in the dystonic muscles
    a. Strongly agree
    b. Agree
    c. Neither agree nor disagree
    d. Disagree
    e. Strongly disagree

17. Please list any other goals which you feel are relevant to the treatment of people with cervical dystonia.

Treatment techniques – Conservative treatment for impairments

All statements are regarding any patient with cervical dystonia. How much do you agree with the following statements?

When treating a patient with cervical dystonia:

1. It is essential to prescribe active movement exercises for the neck and upper limbs
    a. Strongly agree
    b. Agree
    c. Neither agree nor disagree
    d. Disagree
    e. Strongly disagree

2. It is essential to use active or passive muscle stretches for the neck, upper limbs and back
    a. Strongly agree
    b. Agree
    c. Neither agree nor disagree
    d. Disagree
    e. Strongly disagree

3. It is essential to prescribe strengthening exercises for neck, upper back and upper limb muscles
    a. Strongly agree
    b. Agree
    c. Neither agree nor disagree
    d. Disagree
    e. Strongly disagree

4. It is essential to use massage
    a. Strongly agree
    b. Agree
    c. Neither agree nor disagree
    d. Disagree
    e. Strongly disagree

5. It is essential to use passive joint mobilisation of the cervical spine
    a. Strongly agree
    b. Agree
    c. Neither agree nor disagree
    d. Disagree
    e. Strongly disagree

6. It is essential to give advice and prescribe exercises to improve posture and trunk muscle strength
    a. Strongly agree
    b. Agree
    c. Neither agree nor disagree
    d. Disagree
    e. Strongly disagree

7. It is essential to use heat therapy (hot pack)
    a. Strongly agree
    b. Agree
    c. Neither agree nor disagree
    d. Disagree
    e. Strongly disagree

8. It is essential to use cold therapy (eg. ice pack)
    a. Strongly agree
    b. Agree
    c. Neither agree nor disagree
    d. Disagree
    e. Strongly disagree

9. It is essential to use electrophysical agents (e.g. TENS, biofeedback, FES etc)
    a. Strongly agree
    b. Agree
    c. Neither agree nor disagree
    d. Disagree
    e. Strongly disagree

10. It is essential to use Kinesio taping
    a. Strongly agree
    b. Agree
    c. Neither agree nor disagree
    d. Disagree
    e. Strongly disagree

11. It is essential to teach and practise relaxation techniques (eg. Progressive muscle relaxation)
    a. Strongly agree
    b. Agree
    c. Neither agree nor disagree
    d. Disagree
    e. Strongly disagree

Treatment techniques – Education and activity limitations

12. It is essential to give advice on increasing participation in general physical activity e.g. swimming, walking, playing sport etc.
    a. Strongly agree
    b. Agree
    c. Neither agree nor disagree
    d. Disagree
    e. Strongly disagree

13. It is essential to re-train gait / walking
    a. Strongly agree
    b. Agree
    c. Neither agree nor disagree
    d. Disagree
    e. Strongly disagree

14. It is essential to prescribe balance exercises
    a. Strongly agree
    b. Agree
    c. Neither agree nor disagree
    d. Disagree
    e. Strongly disagree

15. It is essential to prescribe exercises to improve coordination and body perception
    a. Strongly agree
    b. Agree
    c. Neither agree nor disagree
    d. Disagree
    e. Strongly disagree

16. It is essential to prescribe breathing exercises
    a. Strongly agree
    b. Agree
    c. Neither agree nor disagree

d. Disagree
e. Strongly disagree

17. It is essential to prescribe Tai Chi, Yoga or Pilates exercises
    a. Strongly agree
    b. Agree
    c. Neither agree nor disagree
    d. Disagree
    e. Strongly disagree

18. It is essential to give advice on mindfulness techniques
    a. Strongly agree
    b. Agree
    c. Neither agree nor disagree
    d. Disagree
    e. Strongly disagree

19. It is essential to have patients participating in group exercise
    a. Strongly agree
    b. Agree
    c. Neither agree nor disagree
    d. Disagree
    e. Strongly disagree

20. It is essential to prescribe vestibular retraining
    a. Strongly agree
    b. Agree
    c. Neither agree nor disagree
    d. Disagree
    e. Strongly disagree

21. It is essential to use visual tracking tasks
    a. Strongly agree
    b. Agree
    c. Neither agree nor disagree
    d. Disagree
    e. Strongly disagree

22. It is essential to retrain upper limb movement and function
    a. Strongly agree
    b. Agree
    c. Neither agree nor disagree
    d. Disagree

e. Strongly disagree

23. It is essential to use sensory retraining
    a. Strongly agree
    b. Agree
    c. Neither agree nor disagree
    d. Disagree
    e. Strongly disagree

24. It is essential to prescribe assistive devices (eg. Neck brace or collar, orthosis or assistive devices for activities of daily living etc.)
    a. Strongly agree
    b. Agree
    c. Neither agree nor disagree
    d. Disagree
    e. Strongly disagree

25. It is essential to teach patients to use sensory tricks to relieve involuntary head turning
    a. Strongly agree
    b. Agree
    c. Neither agree nor disagree
    d. Disagree
    e. Strongly disagree

26. It is essential to use hydrotherapy
    a. Strongly agree
    b. Agree
    c. Neither agree nor disagree
    d. Disagree
    e. Strongly disagree

27. It is essential to provide education on cervical dystonia
    a. Strongly agree
    b. Agree
    c. Neither agree nor disagree
    d. Disagree
    e. Strongly disagree

28. It is essential to use Transcranial Magnetic Stimulation (non-invasive brain stimulation) to treat cervical dystonia
    a. Strongly agree
    b. Agree
    c. Neither agree nor disagree

    d. Disagree
    e. Strongly disagree

29. Please list any other treatment techniques that you think are essential in the treatment of people with cervical dystonia.

Evidence based Practice

30. Are you aware of the current evidence supporting the physiotherapy techniques you use in your treatment? Y / N / unsure

31. Do you use this evidence in your practice? Y/N/unsure

Timing of treatment

32. If patients are having botulinum neurotoxin treatment, what is the ideal time to start physiotherapy (in relation to the application of botulinum neurotoxin injections application)?
    a. Immediately following the injection
    b. Within 1 week of the injection
    c. 1 - 2 weeks after the injection
    d. 3 – 4 weeks after the injection
    e. More than 4 weeks after the injection
    f. It does not matter when physiotherapy is commenced

33. How frequently do you see a patient with cervical dystonia having physiotherapy treatment?
    a. Every day
    b. 2 – 3 times per week
    c. Once per week
    d. Once per fortnight
    e. Once per month
    f. Less often than once per month
34. On average, what are the total number of treatment sessions you provide for a patient with cervical dystonia?
    a. One
    b. 2 – 5
    c. 6 – 10
    d. More than 11
35. How often do you assess your patients using objective measures?
    a. Weekly
    b. Once every 2 weeks
    c. Once per month

d. Once every 3 months
e. At the start and end of treatment only

36. Do your patients have access to other allied health professionals who might be helpful in the management of their cervical dystonia (eg psychologist, occupational therapist, speech therapist)? Y / N / unsure – please list other professionals you have referred your patients to

Online Resource 2: Results from round 1 survey

Questions that have achieved at least 70% agreement:

When assessing a person with iCD, it is essential to include the following:

1. Observe/assess the dystonic position of the head (head turn) and muscle over-activity; measured by observation and palpation.
2. Observe/assess/identify which muscles are dystonic (over-active); measured by palpation, AROM/PROM and observation.
3. Observe the presence/absence of head or limb tremor; measured by observation.
4. assess the patient's ability to actively correct their head position and maintain the aligned position for a period of time
5. Assess the patient's pain due to cervical dystonia; measured by VAS.
6. Assess neck and upper limb ACTIVE range of movement; measured by observation.
7. Assess neck and upper limb PASSIVE range of motion; measured by observation.
8. Measure neck, upper limb and lower limb muscle strength; no consensus on how to measure.
9. Observe the posture of the neck and spine; measured by observation.
10. Assess their ability to coordinate movements of their head, neck and upper limbs; no consensus on how to measure.
11. Assess vision; no consensus on how to measure.
12. Assess balance; no consensus on how to measure.
13. Assess walking speed and ability; no consensus on how to measure.
14. Assess recent falls and ongoing falls risk; no consensus on how to measure.
15. Exercise tolerance and participation in regular exercise; no consensus on how to measure.
16. Assess difficulty working or performing self-care and household tasks (for example, personal hygiene, house and garden chores, driving, working etc); no consensus on how to measure.
17. Ask about neurotoxin use (for example, the neurotoxin type, dosage, frequency of injection and effect).
18. Ask about other medical treatments used to manage their dystonia (for example oral medication, deep brain stimulation).
19. Ask about other medical conditions which may impact on their cervical dystonia.
20. To measure cervical dystonia severity using a standardized scale, for example the Toronto Western Spasmodic Torticollis Rating Scale (TWSTRS) or TSUI scale; no consensus on which scale to use.
21. Use a standardized scale to measure the impact of cervical dystonia on a person's quality of life (CDQ-24, CDIP-58 etc); no consensus on which scale to use.

Measurement tools for assessment that have achieved at least 70% agreement:

1. Observation
2. Palpation
3. AROM/PROM – measured by observation
4. Visual Analogue Scale (VAS) for pain
5. Posture of the neck and spine – measured by observation

The goals of physiotherapy that have achieved at least 70% agreement:

1. To improve the patient's active head control at rest and during movement.
2. To improve or reduce head, neck or upper limb tremor.
3. To reduce the pain related to cervical dystonia.
4. To improve the patient's sensorimotor integration.
5. To improve or increase active range of movement of the neck.
6. To improve strength in the muscles of the neck and upper back.
7. To improve strength, coordination and function of the upper limbs.
8. To improve general fitness and increase participation in general exercise.
9. To improve the patient's balance.
10. To improve walking function (for example gait pattern, speed, safety).
11. To teach self-management strategies for cervical dystonia.

Treatment techniques that have achieved at least 70% agreement:

1. Active movement exercises for the neck and/or upper limbs – 100% agreement.
2. Passive muscle stretches for the neck, upper limbs and back.
3. Strengthening exercises for neck, upper back and upper limb – 100% agreement.
4. Give advice and prescribe exercises to improve posture – 100% agreement.
5. To teach/prescribe and practise relaxation techniques (eg. Progressive muscle relaxation) – 100% agreement.
6. To encourage increasing participation in general physical activity e.g. swimming, walking, playing sport etc – 100% agreement.
7. Re-train gait / walking.
8. Prescribe balance exercises.
9. Prescribe exercises to improve coordination and body perception.
10. Prescribe breathing exercises.
11. To give advice on meditation and/or mindfulness techniques.
12. Prescribe/ practise visual tracking tasks.
13. Re-train upper limb movement and function.
14. Sensory retraining.
15. To provide education on cervical dystonia – 100% agreement.
16. Massage and the use of cold packs were not supported as treatment techniques by more than 70% of participants and so should not be recommended as treatment techniques.
17. The use of assistive devices (including neck braces) was not supported by 60% of participants and the remaining participants held a neutral view. In this case, should these not be recommended?

Evidence based practice:

89% of participants reported that they have an awareness of and are using the current evidence supporting the physiotherapy techniques in the treatment of iCD.

80% of participants reported that they have access to other allied health professionals who might be helpful in the management of their patients with cervical dystonia (eg psychologist, occupational therapist, speech therapist, exercise physiologist).

Number, timing and frequency of treatments:

There was no consensus among participants on the frequency, duration or timing of treatment sessions for people with iCD.

Online Resource 3: Round 2 survey questions

Please review the demographic information of participants, the complete results (in tables 1-4) and the summary document of items that have achieved at least 70% consensus among participants of round 1 of the Delphi process. Then proceed to the next survey (below).

1. To complete the demographic information of participants, we would like to ask one extra question: Please indicate the percentage of your workload that is focussed on researching or treating patients with iCD?

The following questions have been rated by participants in round 1 as essential to include in the assessment of people with iCD. Due to the large number of items, it is unlikely that all of these items would be assessed for every patient. With this in mind, could you please rank each assessment item as either "ESSENTIAL" to assess in every iCD patient or "OPTIONAL" to assess in patients with iCD if required.

Assessment of patients with iCD:

22. Observe/assess the dystonic position of the head (head turn) and muscle over-activity Essential / Optional
23. Observe/assess/identify which muscles are dystonic (over-active) Essential / Optional
24. Observe the presence/absence of head or limb tremor Essential / Optional
25. Assess the patient's ability to actively correct their head position and maintain the aligned position for a period of time Essential / Optional
26. Assess the patient's pain due to cervical dystonia Essential / Optional
27. Assess neck and upper limb ACTIVE range of movement Essential / Optional
28. Assess neck and upper limb PASSIVE range of motion Essential / Optional
29. Measure neck, upper limb and lower limb muscle strength Essential / Optional
30. Observe the posture of the neck and spine Essential / Optional
31. Assess their ability to coordinate movements of their head, neck and upper limbs Essential / Optional
32. Assess vision Essential / Optional

Activity limitations/participation restrictions:

33. Assess balance Essential / Optional
34. Assess walking speed and ability Essential / Optional
35. Assess recent falls and ongoing falls risk Essential / Optional
36. Exercise tolerance and participation in regular exercise Essential / Optional
37. Assess difficulty working or performing self-care and household tasks (for example, personal hygiene, house and garden chores, driving, working etc) Essential / Optional
38. Ask about neurotoxin use (for example, the neurotoxin type, dosage, frequency of injection and effect). Essential / Optional
39. Ask about other medical treatments used to manage their dystonia (for example oral medication, deep brain stimulation). Essential / Optional

40. Ask about other medical conditions which may impact on their cervical dystonia. Essential / Optional
41. To measure cervical dystonia severity using a standardized scale, for example the Toronto Western Spasmodic Torticollis Rating Scale (TWSTRS) or TSUI scale Essential / Optional; which scale would you prefer to use – TWSTRS OR TSUI?
42. Use a standardized scale to measure the impact of cervical dystonia on a person's quality of life (CDQ-24, CDIP-58 etc) Essential / Optional – which scale would you prefer to use – CDQ-24 OR CDIP-58?

Please refer to table 1 for the next question. Are there any items in Table 1 (Assessment) that did not achieve 70% consensus that you believe are essential or optional to assess in a person with iCD? (Y/N) Please indicate which items and explain why you want them to be included as essential or optional in the final report.

Measurement tools

The following measurement tools were rated as essential by at least 70% of participants. Please indicate whether each tool is "ESSENTIAL" to use in every patient with iCD or "OPTIONAL" to use as required.

1. Observation Essential / Optional
2. Palpation Essential / Optional
3. Visual Analogue Scale (VAS) for pain Essential / Optional

VAS is the only objective measure noted by at least 70% of participants to be used in the management of people with iCD. Please list any other OBJECTIVE measures (i.e. measures that give a numerical score) that you use for patients with iCD.

Goals of physiotherapy in the management of iCD

The following goals of physiotherapy were deemed essential in the management of people with iCD by at least 70% of participants.

Please rank these goals in order of importance from 1 (most commonly used goals in your treatment of iCD) to 10 (least commonly used goals in your treatment of iCD).

12. To improve the patient's active head control at rest and during movement. 1-10
13. To improve or reduce head, neck or upper limb tremor. 1-10
14. To reduce the pain related to cervical dystonia. 1-10
15. To improve the patient's sensorimotor integration. 1-10
16. To improve or increase active range of movement of the neck. 1-10
17. To improve strength in the muscles of the neck and upper back. 1-10
18. To improve strength, coordination and function of the upper limbs. 1-10
19. To improve general fitness and increase participation in general exercise. 1-10

20. To improve the patient's balance. 1-10
21. To improve walking function (for example gait pattern, speed, safety). 1-10
22. To teach self-management strategies for cervical dystonia. 1-10

Is it necessary to ask the patient to nominate goals that are specific to them (personal goals addressing individual issues related to iCD)? Y/N

If yes, is this ESSENTIAL for every iCD patient or OPTIONAL for iCD patients as required?

Please refer to table 2 for the next question. Are there any items in Table 2 (Goals of treatment) that did not achieve 70% consensus that you believe are essential or optional to assess in a person with iCD? (Y/N) Please indicate which items and explain why you want them to be included as essential or optional in the final report.

Treatment techniques for iCD

The items below were rated as essential in the physiotherapy treatment of iCD by at least 70% of participants. Considering the large number of treatments, it is unlikely all treatments would be used for all people with iCD. With this in mind, could you please rank each treatment item as either "ESSENTIAL" to use in every iCD patient or "OPTIONAL" to use in patients with iCD if required.

18. Active movement exercises for the neck and/or upper limbs Essential / Optional
19. Passive muscle stretches for the neck, upper limbs and back Essential / Optional
20. Strengthening exercises for neck, upper back and upper limb Essential / Optional
21. Give advice and prescribe exercises to improve posture Essential / Optional
22. To teach/prescribe and practise relaxation techniques (eg. Progressive muscle relaxation) Essential / Optional
23. To encourage increasing participation in general physical activity e.g. swimming, walking, playing sport etc Essential / Optional
24. Re-train gait / walking Essential / Optional
25. Prescribe balance exercises Essential / Optional
26. Prescribe exercises to improve coordination and body perception Essential / Optional
27. Prescribe breathing exercises Essential / Optional
28. To give advice on meditation and/or mindfulness techniques Essential / Optional
29. Prescribe/ practise visual tracking tasks Essential / Optional
30. Re-train upper limb movement and function Essential / Optional
31. Sensory retraining Essential / Optional
32. To provide education on cervical dystonia Essential / Optional

The items below were rated as NOT essential in the physiotherapy treatment of iCD by at least 70% of participants (that is, most participants disagreed that these items are essential). Could you please rank each treatment item below as either "ESSENTIAL" to use in every iCD patient or "OPTIONAL" to use in patients with iCD if required or "NOT RECOMMENDED" for people with iCD and give a reason for your choice.

1. Use of massage Essential / Optional / Not recommended
2. Use of cold therapy (i.e. cold packs) Essential / Optional / Not recommended

Please refer to table 3 for the next question. Are there any items in Table 3 (Treatment techniques) that did not achieve 70% consensus that you believe are essential or optional to assess in a person with iCD? (Y/N) Please indicate which items and explain why you want them to be included as essential or optional in the final report.

General questions:

1. Are there any items in Table 4 regarding frequency and duration of treatment that did not achieve 70% consensus that you believe are essential or optional to include in the final report? (Y/N) Please indicate which items and explain why you want them to be included as essential or optional in the final report.

2. As there was no consensus regarding recommended number of treatments, frequency of treatment or when to commence physiotherapy, do you believe that these decisions should be made by the physiotherapist and patient on a case by case basis? (Y/N) Please give a reason for your response.

3. To account for differences in physiotherapy practice internationally, when assessing functional tasks such as walking, balance, exercise tolerance, falls risk etc, is it acceptable to use any standardised, validated assessment scale for each task? (Y/N) Please give a reason.

Online Resource 4: Results from round 2 survey

Survey 2 was completed by 9 participants, all of whom completed the first survey. One participant completed survey 1 but did not complete survey 2. Participants were physiotherapists from Australia, the United Kingdom, the Netherlands, France, Belgium and the USA who have experience working with people with cervical dystonia. The percentage of time participants work with people with cervical dystonia ranged from 1% to 100% with an average of 40% of their working week. Consensus was reached among participants when at least 70% of them agreed with the statement.

Physiotherapy assessment of a person with cervical dystonia

The following items **have achieved consensus** among participants and will be included in the manuscript.

Essential assessments to perform when assessing a person with cervical dystonia:

1. Assess / ask about pain due to cervical dystonia
2. Assess / ask about difficulty of working or performing self-care and household tasks (for example, personal hygiene, house and garden chores, driving, working etc) with cervical dystonia
3. Ask about neurotoxin use (for example, the neurotoxin type, dosage, frequency of injection and effect)
4. Ask about other medical treatments used to manage cervical dystonia (for example oral medication, deep brain stimulation)
5. Ask about other medical conditions which may impact on cervical dystonia
6. Observe/assess the dystonic position of the head (head turn) and muscle over-activity
7. Observe/assess/identify which muscles are dystonic (over-active)
8. Observe the presence/absence of head or limb tremor
9. Observe posture of the neck and spine
10. Assess the ability to actively correct head position and maintain the aligned position for a period of time
11. Assess neck and upper limb active range of movement (AROM)
12. Assess ability to coordinate movements of the head, neck and upper limbs

The following additional assessments were considered essential to include in an assessment by more than 70% of participants in the round 1 survey. However, there was no consensus among participants in the round 2 survey as to whether they are essential to perform on all people with cervical dystonia or optional to perform as required. **They will therefore be listed as optional in the manuscript.**

1. Assess neck and upper limb passive range of motion
2. Measure neck, upper and lower limb muscle strength
3. Assess balance
4. Assess walking speed and ability

5. Assess any recent falls and ongoing falls risk
6. Assess exercise tolerance and participation in regular exercise
7. Assess vision

Measurement

Consensus was achieved that the following measurements are **essential** to include when assessing a person with cervical dystonia. These will be included in the manuscript.

1. Observation
2. Visual Analogue Scale (VAS) for pain

The following additional measurements were considered essential to include in an assessment by more than 70% of participants in the round 1 survey. However, there was no consensus among participants in the round 2 survey as to whether they are essential to perform on all people with cervical dystonia or optional to perform as required. **They will therefore be listed as optional in the manuscript.**

1. Palpation of the neck, shoulders and upper back
2. Measure cervical dystonia severity using a standardized scale, for example the Toronto Western Spasmodic Torticollis Rating Scale (TWSTRS) or TSUI scale
3. Measure the impact of cervical dystonia on a person's quality of life using a standardised scale, for example the CDQ-24 or CDIP-58

Other measurement tools suggested by participants that did not reach 70% consensus on their use for cervical dystonia were:

- Functional gait assessment (FGA)
- Static standing balance time
- Degrees of active and passive neck range of motion (instrument used to measure was not specified)
- Video analysis, inertial sensors, sensamove, 3D neck movement analysis using Conebeam or scanners.
- Clinical Global Impressions Severity (CGI-s) and improvement (CGI-I)
- Symmetry index
  MDS Non motor scale

These will not be included in the manuscript.

Goals of physiotherapy

All participants agreed that it is essential to discuss specific, individual goals of physiotherapy with the person with cervical dystonia. The physiotherapist and the person with cervical dystonia should collaborate to devise an individualised treatment plan which addresses specific goals and encourages self-management.

In addition, the following goals of physiotherapy for people with cervical dystonia were determined by consensus of participants to be **essential** and will be included in the manuscript. The participants have ranked these goals in order of importance from top to bottom (ie. no. 1 is the most important for people with cervical dystonia). When devising individual treatment goals with a person with cervical dystonia, the physiotherapist should consider the goals listed below.

1. To teach self-management strategies for cervical dystonia
2. To improve active head control at rest and during movement
3. To reduce pain related to cervical dystonia
4. To improve / increase active range of movement of the neck
5. To improve sensorimotor integration
6. To improve general fitness and increase participation in general exercise
7. To improve strength in the muscles of the neck and upper back
8. To improve / reduce head, neck or upper limb tremor
9. To improve strength, coordination and function of the upper limbs
10. To improve walking function (for example gait pattern, speed, safety)
11. To improve balance

The following suggested additional goals of physiotherapy did not achieve 70% consensus among participants and so will not be listed in the manuscript.

- To improve sleep
- To improve the ability to perform work-related tasks or activities of daily living
- To understand the mechanism of dystonia including aggravating and easing factors

Physiotherapy treatment techniques for cervical dystonia

Consensus was reached among participants that the following techniques are **essential** to include in physiotherapy treatment sessions. These will be included in the manuscript.

1. Education on cervical dystonia
2. Active movement exercises for the neck and/or upper limbs
3. Exercises and education to improve posture
4. Relaxation techniques (eg. Progressive muscle relaxation)
5. Increasing participation in general physical activity (for example swimming, walking, playing sport etc)
6. Sensory retraining
7. Other treatment techniques that address specific individual goals of the person with cervical dystonia

Consensus was reached between participants that the following techniques are **optional** to include in physiotherapy treatment sessions as required:

1. Education on meditation and/or mindfulness techniques
2. Exercises to improve upper limb movement and function
3. Breathing exercises

The following additional techniques were considered essential to include in an assessment by more than 70% of participants in the round 1 survey. However, there was no consensus among participants in the round 2 survey as to whether they are essential to perform on all people with cervical dystonia or optional to perform as required. **They will therefore be listed as optional in the manuscript.**

1. Passive muscle stretches for the neck, upper limbs and back
2. Strengthening exercises for neck, upper back and upper limb
3. Gait / walking retraining
4. Balance exercises / re-training
5. Exercises to improve coordination and body perception
6. Visual tracking tasks / exercises

Other treatment techniques suggested by participants that did not reach 70% consensus on their use for cervical dystonia were:

- Education on sensory tricks and their use to reduce involuntary movements
- Biofeedback and visual feedback in exercise tasks
- Neuromodulation methods combined with motor practice, e.g. anodal tDCS of cerebellum combined with goal-oriented motor training
- Distraction techniques

These techniques will not form part of the manuscript.

Treatments which may cause harm to people with cervical dystonia

Consensus was reached among participants that the following techniques were **not recommended** to be included in treatment sessions with people with cervical dystonia as they could lead to worsening symptoms:

1. Cold / ice packs

Therefore, this will be listed as not recommended in the manuscript.

Consensus was not reached between participants whether the following techniques should be listed as **not recommended or optional** for use during therapy. These techniques could trigger an increase in cervical dystonia symptoms but could also assist in pain control. These techniques should be used with caution and the effects on individuals should be monitored closely.

1. Massage to the neck, shoulders and upper back

Therefore, this will be listed as optional in the manuscript, with a warning to monitor the effects on each person closely during therapy.

General management

Consensus was achieved among participants that frequency, duration and timing of physiotherapy treatment should be determined on a case-by-case basis depending on duration and severity of symptoms, the person's goals for therapy and their response to treatment.

Consensus was achieved among participants that is it acceptable to use any standardised, validated assessment scale to measure functional tasks (such as walking and balance) in people with cervical dystonia. These general management principles will be included in the manuscript.
